# Transmission Potential and Design of Adequate Control Measures for Marburg Hemorrhagic Fever

**DOI:** 10.1371/journal.pone.0050948

**Published:** 2012-12-10

**Authors:** Marco Ajelli, Stefano Merler

**Affiliations:** Bruno Kessler Foundation, Trento, Italy; Tulane School of Public Health and Tropical Medicine, United States of America

## Abstract

Marburg hemorrhagic fever is rare yet among the most severe diseases affecting humans, with case fatality ratio even higher than 80%. By analyzing the largest documented Marburg hemorrhagic fever epidemic, which occurred in Angola in 2005 and caused 329 deaths, and data on viral load over time in non-human primates, we make an assessment of transmissibility and severity of the disease. We also give insight into the control of new Marburg hemorrhagic fever epidemics to inform appropriate health responses. We estimated the distribution of the generation time to have mean 9 days (95%CI: 8.2–10 days) and standard deviation 5.4 days (95%CI: 3.9–8.6 days), and the basic reproduction number to be 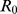 = 1.59 (95%CI: 1.53–1.66). Model simulations suggest that a timely isolation of cases, starting no later than 2–3 days after symptoms onset, is sufficient to contain an outbreak. Our analysis reveals that Marburg hemorrhagic fever is characterized by a relatively small reproduction number and by a relatively long generation time. Such factors, along with the extremely high severity and fatality, support the rare occurrence of large epidemics in human populations. Our results also support the effectiveness of social distancing measures - case isolation in particular - to contain or at least to mitigate an emerging outbreak. This work represents an advance in the knowledge required to manage a potential Marburg hemorrhagic fever epidemic.

## Introduction

Marburg hemorrhagic fever is a viral hemorrhagic fever caused by Marburg virus (MARV), which belongs to the family of filoviruses along with Ebola virus. After the original MARV epidemic in West Germany and Yugoslavia in 1967 [1], sporadic cases were reported in Kenya [2], [3], while epidemic outbreaks were observed in South Africa [4], Democratic Republic of the Congo [5], [6] and Angola, where the most devastating epidemic took place in 2005 [7], [8]. In recent years, one imported case has also been reported in Colorado, USA [9] and one in The Netherlands [10]. Despite the relatively low number of outbreaks in human populations, MARV has largely been identified in both fruit (*Rousettus aegyptiacus*) and insectivorous (*Rhinolophus eloquens* and *Miniopterus inflatus*) bats in several areas of Africa [11]–[13]. In addition, in 2011 a new genetically distinct filovirus has been discovered in dead insectivorous bats in Spain [14]. MARV transmission occurs through direct contact between humans; however, it is thought to occur also by handling ill or dead infected animals (mainly monkeys and bats) and human corpses [7]. MARV is among the most virulent pathogens infecting humans, with case fatality ratio (CFR) even higher than 80% in two recent outbreaks in the Democratic Republic of the Congo and Angola [15]. Despite several studies focus on the development of vaccines and therapies for MARV (see for instance [16], [17]; a recent review can be found in [18]), neither an effective vaccine nor a treatment for human infections is currently available. In fact, MARV has all the key features that characterize pathogens posing serious risks for human populations if used as biological weapons [19].

For the previously mentioned reasons, a possible MARV epidemic would represent a serious threat for human health and would pose lots of questions to policy makers in the management of an outbreak. Consequently, a deeper knowledge of the main epidemiological determinants of MARV epidemics is crucial to plan adequate control measures. This study aims to provide estimates of generation time distribution and transmission potential and, through the use of a mathematical simulation model, to assess the effectiveness of social distancing measures in order to inform appropriate health responses in case of future MARV epidemics.

## Materials and Methods

### MARV Natural History and Description of the Analyzed Data

Marburg hemorrhagic fever presents as an acute febrile illness which usually progresses to severe hemorrhagic manifestations. The incubation period is followed by a sudden symptoms onset marked by fever, chills, headache, and myalgia. After that a maculopapular rash may manifest, and the individual may experience nausea, vomiting, chest pain, sore throat, abdominal pain, and/or diarrhea. The progress of the disease is accompanied by increasingly severe symptoms and patients often develop severe hemorrhagic manifestations. The final stages of the disease include inflammation of the pancreas, severe weight loss, delirium, shock, liver failure, and multiorgan dysfunction - fatal cases usually have some form of bleeding, often from multiple sites [7], [15].

As reported by the Global Alert and Response updates of the WHO [15], the 2005 epidemic in Angola was the largest MARV outbreak documented so far. Since MARV was identified as the virus responsible for the outbreak by the CDC laboratories on March 25, 2005, case count was based on the application of clinical case definition, later supported by on-site laboratories. However, a retrospective analysis showed that the outbreak probably started in October 2004. The epidemic accounted for 374 reported cases of which 329 resulted fatal (CFR = 88%) and spread almost only in Uige region (Northern Angola) [15], which accounts for about 500,000 individuals [20]. Approximately 75% of the first 124 identified cases occurred in children aged 5 years or younger [15]: in 2005, this age group accounted for 26.4% of the total population of Angola [21], which is characterized by a low average age and a high fertility rate. The epidemic was declared over by the Angolan Ministry of Health on November 9, 2005 [15].

As pointed out in the literature [22]–[24], the shape of the generation time distribution is essential to evaluate the effectiveness of individually targeted control measures (e.g. case isolation). To such aim, we complemented our investigation of the 2005 epidemic by performing a new analysis of the experimental results on viral load data over time in non-human primates (specifically on *Cercopithecus aethiops*) injected by Marburg virus, as reported in [25].

### Estimation Procedure for the Generation Time

The generation time (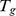) is defined as the duration between the time of infection of a secondary case and the time of infection of its primary infector. This is equivalent in length to the serial interval, which represents the duration between the time of symptoms onset of a secondary case and the time of symptoms onset of its primary infector.

The distribution of 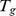 is strongly related to the infectiousness over time of infected individuals. We assume a direct proportion between viral load and infectiousness, as already suspected for MARV [26]; thus we model the latter as a function 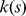 depending on the time elapsed from the end of the latent period 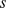. This assumption, largely adopted in the literature (see e.g., [22]–[24]), is a more biologically sound hypothesis than assuming constant infectiousness over the entire infectivity period. The average generation time is given by the mean latent period plus 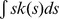.

By fitting a gamma distribution with offset (to account for the latent period) to the average viral load over time since the time of infection [25], we obtain latent period estimate of 3 days, average 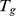 estimate of 13.9 days (95%CI: 12.7–17 days) and standard deviation 7.5 days (95%CI: 5.5–11.4 days). The above 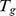 estimates, however, do not account for disease related mortality which can not be disregarded to obtain reliable estimates for highly lethal diseases, like Marburg hemorragic fever. To correct 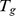 estimates, we adopt the following procedure: first, by randomly sampling from the observed values of viral load over time, we generate different individual profiles of infectiousness over time (by fitting a gamma distribution with offset); second, we assume that only the fraction 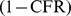 of MARV infected survive to the peak of the viral load, and thus we weigh the values of the decaying phase of the infectiousness profiles over time by the factor 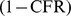. Finally, the resulting average infectiousness profile is normalized in such a way that the sum of all elements is equal to one: this corresponds to the probability density distribution of 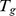.

### Estimation Procedure for the Basic Reproduction Number

The basic reproduction number (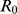) is defined as the number of new infections generated by one infective individual during the entire period of infectiousness in a fully susceptible population [27].


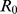
 for the 2005 outbreak in Angola can be estimated as 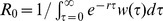, where 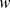 is the probability density distribution of 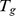 and 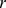 is the exponential growth rate of the epidemic, i.e. the growth rate of the cumulative number of MARV infections observed during the early phases of the 2005 epidemic in Angola, when no intervention measures were enacted and the depletion of susceptible individuals was negligible.

Details on the derivation of the equation for 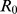 can be found in [28]. The same technique was already applied, for instance, to the analysis of epidemic outbreaks caused by Ebola virus [29], for the 2009 H1N1 influenza pandemic [30], [31], for historical influenza records [23], [32], [33] and for the analysis of the output of model simulations [22], [24], [34], [35].

### Simulation Model

We propose a mathematical simulation model to evaluate the effectiveness of different containing/mitigation measures for MARV epidemics. The adopted model is a discrete time stochastic Markov chain, where individuals are explicitly represented to account for individual variability of infectiousness over time.

The possible epidemiological status of an individual is: susceptible, infected, recovered and dead. At each time step of the simulation 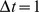 day, each susceptible individual 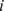 is exposed to the same force of infection 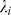 and has a probability 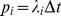 of becoming infected; the force of infection at time 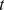 can be written as 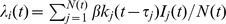, where 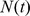 is the number of (alive) individuals at time 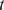; 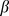 is the transmission rate; 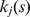 is the infectiousness of individual 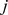, 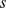 days after infection; 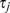 is the time at which individual 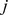 became infected; 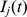 is 1 if individual 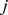 is infectious (not isolated, see next section) at time 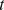, 0 otherwise.

An individual infectiousness profile over time 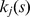 is assigned to each infected individual by randomly choosing from the different individual infectiousness profiles as obtained by randomly sampling from the fitted values of viral load over time. When an individual reaches the peak of her/his infectiousness, she/he has a probability of death equal to the CFR. Therefore, the individual can either be removed from the simulation or to progress to the recovered class when her/his infectiousness approaches zero.

### Description of Social Distancing Strategies

The main social distancing measure considered in this study is the isolation of infective cases. Specifically, we assume that each infected individual is not isolated for a certain time since symptoms onset (incubation and latent periods are assumed to coincide, thus symptoms onset coincides with the start of infectiousness), and thereafter she/he has a daily probability of becoming isolated. When isolated, individuals are assumed to not contribute to the force of infection. We perform a sensitivity analysis by varying: 1) first isolation day, that is the minimum time elapsed from symptoms onset to the first possible isolation day; it accounts for the time required to recognize a MARV case and to take the appropriate decisions; 2) daily isolation probability, which accounts for both probability of isolating a MARV case and isolation efficacy; 3) the overall number of deaths (caused by MARV infection) in the population before starting the strategy.

We also investigate the effects of another kind of social distancing measure, which involves the whole population. In particular, here we consider the possibility of an intensive social mobilization (as it was the case of the 2005 MARV outbreak in Angola [7]), in order to inform the general population on the routes of transmission of the disease and to promote less risky behaviors, for instance when contacting other individuals, handling dead animals or human corpses. We model this behavioral response of the population by assuming a decrease in the number of potentially infectious contacts and thus a reduction of the force of infection.

## Results

### Generation Time

**Figure 1:**
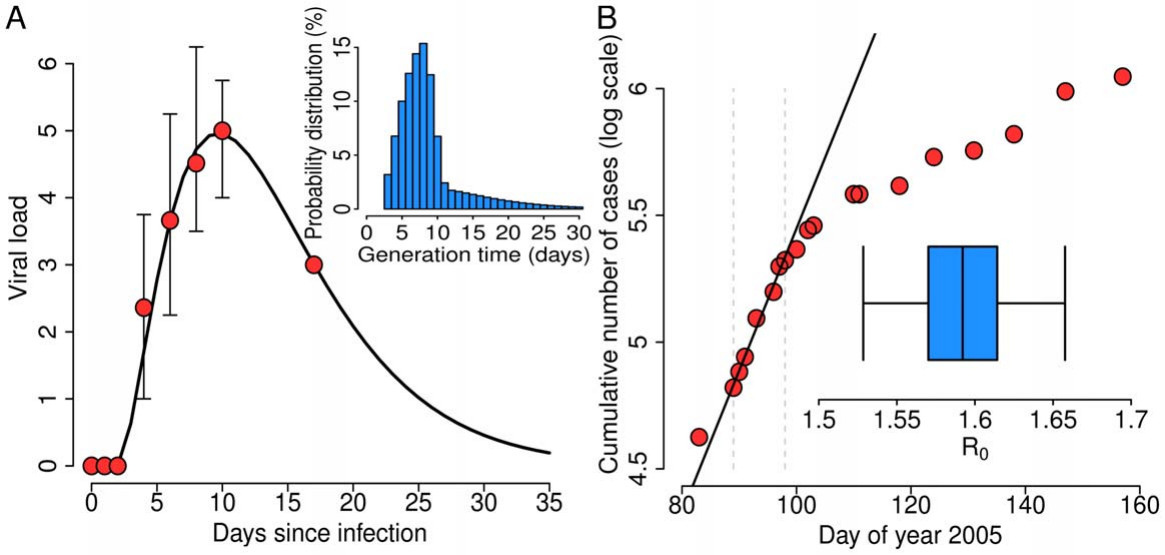
Generation time and reproduction number. **A** Average (red dots) and minimum/maximum (vertical black lines) viral load (measured in IgLD_50_/ml) since the day of infection, as observed in [25]. The black line represents the best fit to the average values. The inset shows the resulting generation time distribution (CFR = 88%). **B** Logarithm of the cumulative number of cases over time (red dots) as reported during the 2005 Marburg hemorrhagic fever epidemic in Angola [15]. The black line represents the best fit of a linear model to the data during the exponential growth phase of the epidemic (delimited in the panel by the vertical dashed lines). The inset shows 2.5% quantile, 25% quantile, mean, 75% quantile and 97.5% quantile of the estimated basic reproduction number distribution.

The best parameters of a gamma distribution fit to data on viral load over time are 2.66 (95% CI: 1.67–4.09) for the shape parameter and 4.78 (95% CI: 2.73–8.85) for the scale parameter; the offset of the distribution is 2 days. A comparison between estimated infectiousness profile and empirical data on viral load over time is shown in Figure 1A. When we take into account the case fatality ratio (88%, as resulting from the analysis of the outbreak in Angola; see Table 1), the estimated average 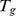 is 9 days (95% CI: 8.2–10 days), with standard deviation of 5.4 days (95% CI: 3.9–8.6 days); the shape of the distribution is shown in the subpanel of Figure 1A. We estimate that MARV infections result in a fatal outcome a median of 7 days (range, 5–9 days) after symptoms onset.

**Table 1:** Epidemiological parameters estimates.

Variable	Estimate (average and 95%CI)	Source of the analyzed data
Case fatality rate	88% (95%CI: 84%–91%)	WHO outbreak report [15]
Doubling time	12.4 days (95%CI: 11.3–13.6 days)	WHO outbreak report [15]
Generation time distribution	Mean 9 days (95%CI: 8.2–10 days)	Experimental study [25]
	SD 5.4 days (95%CI: 3.9–8.6 days)	
Reproduction number	1.59 (95%CI: 1.53–1.66)	WHO outbreak report [15]

Estimated values of case fatality rate, reproduction number and generation time of Marburg virus, and source of empirical data analyzed. CI = Confidence Interval; SD = Standard Deviation.

We also perform an alternative analysis where we assume that death probability is directly proportional to the viral load (instead of assuming death to occur at the peak of viremia). In these new experiments we estimate the generation time to be on average 9.3 days (95% CI: 3.7–14.6 days) and that infections result in a fatal outcome a median of 9 days (range, 0–56 days) after symptoms onset.

### Basic Reproduction Number

MARV notification data in Angola reported to the WHO [15] show a clear exponential growth phase in March-April 2005; we estimate the intrinsic growth rate of the epidemic in that period, when social mobilization was not promoted yet, to be 0.056 days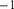 (95% CI: 0.0508–0.0612 days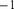), which results in a doubling time of 12.4 days (95% CI: 11.3–13.6 days), see Figure 1B. The resulting estimate of the basic reproduction number is 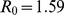 (95% CI: 1.53–1.66); see the inset in Figure 1B. A summary of the estimated epidemiological parameters is reported in Table 1.

### Baseline Scenario

**Figure 2:**
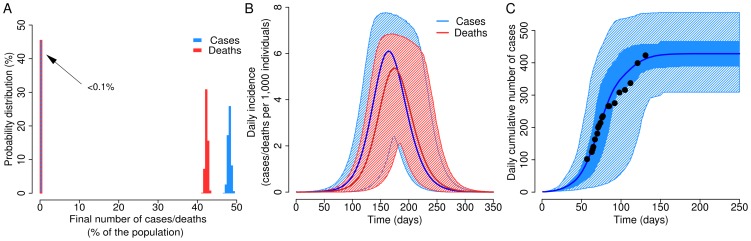
Dynamics of uncontrolled epidemics and the 2005 Marburg hemorrhagic fever epidemic in Angola. **A** Predicted probability distribution of the final number of cases (blue) and deaths (red) as percentage of the population in uncontrolled epidemics. In 45.1% of model simulations both final number of cases and deaths are less than 0.1% of the population. **B** Daily incidence of new cases (blue line and dashed area represent mean and 95% CI respectively) and deaths (red line and dashed area represent mean and 95% CI respectively) of simulated uncontrolled epidemics, initialized with one infected individual at time 0 in a population of 100,000 individuals. **C** Cumulative number of Marburg hemorrhagic fever cases as reported in the 2005 epidemic in Angola [15] (black dots) and as resulting from model simulations (blue line, blue shaded area and blue dashed area represents average, 50%CI and 95%CI respectively). Model simulations assume that isolation of cases starts when 120 deaths are observed, daily isolation probability is set to 30%, first isolation day is set to 2 days and the size of the population is 500,000 individuals [20]. Results reported in this figure are based on 10,000 model realizations.

Given our estimates of generation time/infectiousness profile, basic reproduction number, and CFR during the 2005 MARV epidemic in Angola, we are able to simulate the spread of a MARV epidemic. Model simulations reveal a possible devastating impact of an uncontrolled MARV epidemic spreading in a fully susceptible population, with a final attack rate of 48.1% (95% CI: 47.4%–48.9%) of the population and a cumulative number of deaths caused by MARV infections of 42.3% (95% CI: 41.8%–42.9%) of the population; the peak day incidence is expected to be on average 0.776% (95% CI: 0.735%–0.822%) with an average peak day percentage of deaths of 0.685% (95% CI: 0.648%–0.726%). On the other hand, the probability of experiencing an epidemic outbreak when one infected individual is introduced in a fully susceptible population is quite low: in 45.5% of the simulations the final attack rate is much lower than 0.1% of the population; in a population of 100,000 individuals, the peak day would occur on average at day 167 (95% CI: 141–209), while the peak day for the MARV deaths results delayed of a few days: on average it is expected on day 177 (95% CI: 150–220). Such a high variability in the epidemic timing is mainly determined by the high stochasticity in the transmission process in the early phase of the epidemic. Results are shown in Figure 2A and B.

However, such kind of “uncontrolled” epidemics are unlikely: in fact, social distancing measures, either enacted by public health authorities or spontaneously driven by the reaction of the population to an ongoing epidemic, would be performed with a non negligible impact. In particular, as observed during the 2005 MARV outbreak in Angola, after an initial phase characterized by uncontrolled spread in the population, an intensive social mobilization occurred [7]. By assuming a population of 500,000 indviduals, as in Uige region [20], the agreement between model simulations and the data on the 2005 MARV epidemic is excellent in the early, exponentially growing, phase of the outbreak (see Figure 2C). Subsequently, a sudden decline in the growth rate is reported in the data, probably determined by the enacted social mobilization [7], uncompliant with model simulations of uncontrolled epidemics. By assuming isolation of cases (see Figure 2C), model simulations are in good agreement with the observed data for the whole course of the epidemic – all data lie in the 50% CI of model predictions. We remark that parameter values used to produce results shown in Figure 2C are merely illustrative, as several sets of parameters lead to a satisfactory model fit.

### Social Distancing Strategies

**Figure 3:**
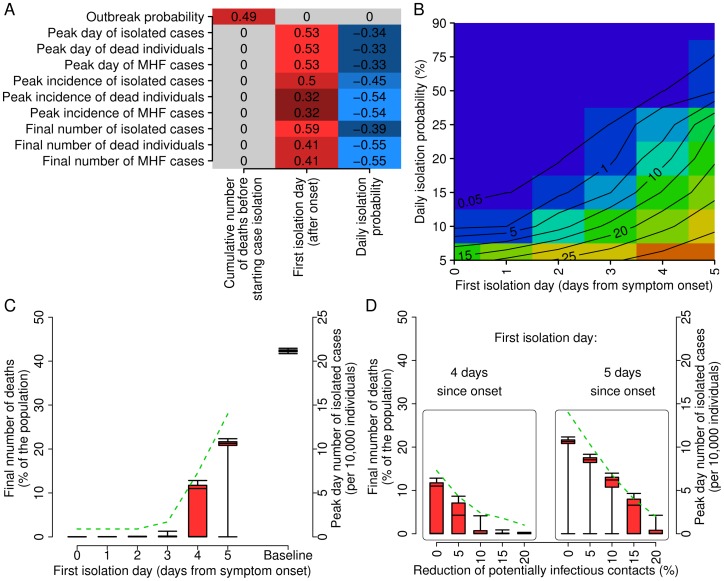
Effectiveness of social distancing strategies. **A** Correlation (computed as Pearson correlation coefficient) between epidemiologically relevant quantities and parameters regulating the implemented case isolation strategy. Values equal to zero means that no statistically significant correlation (p-value 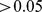) was found. Color scale (from dark blue to light red) reflects the reported correlation values. Parameter space exploration was performed by sampling 500 parameter sets in the following ranges: cumulative number of Marburg hemorrhagic fever deaths before starting case isolation in 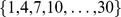, first isolation day in 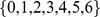 and daily isolation probability sampled from a uniform distribution 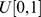. **B** Final number of deaths (as percentage of the population) as a function of the first isolation day (in days from symptoms onset) and of the daily isolation probability (in percentage). Colors from blue to red represent a final number of deaths less than 0.05%, 1%, 5%, 10%, 15%, 20%, 25%, 30% and 35% of the total population. Simulations assume that the isolation of cases starts when 3 deaths per 100,000 individuals are observed in the population. **C** Probability distribution (2.5%, 25%, 50%, 75% and 97.5% quantiles) of the final number of deaths (in percentage of the population, scale on left axis) as a function of the first isolation day. The green dashed line represents the average peak day incidence of isolated individuals (per 100,000 individuals, scale on the right). Simulations assume that the isolation of cases starts when 3 deaths per 100,000 individuals are observed in the population, and the daily isolation probability is kept fixed to 20%. **D** Probability distribution (2.5%, 25%, 50%, 75% and 97.5% quantiles) of the final number of deaths (in percentage of the population, scale on left axis) as a function of the reduction of potentially infectious contact. The green dashed lines represent the average peak day incidence of isolated individuals (per 100,000 individuals, scale on the right). Simulations assume that the isolation of cases starts when 3 deaths per 100,000 individuals are observed in the population, and the daily isolation probability is kept fixed to 20%; the first isolation day is set to 4 days in the left box and to 5 days in right one. Results reported in this figure are based on 10,000 model realizations.

The effects of simulated social distancing measures depend on three parameters: first isolation day, daily isolation probability, and cumulative number of MARV deaths in the population before starting to perform the strategy. As expected, the overall number of MARV deaths in the population before enacting the case isolation strategy has a statistically significant correlation only with the probability of observing a major outbreak. On the other hand, both daily isolation probability and first isolation day have a significant impact on all other epidemiologically relevant quantities such as, for instance, cumulative number of cases/deaths, peak daily incidence and timing of the epidemic (see Figure 3A). Therefore, we focus our investigation on the evaluation of the effects of these two parameters.

Model simulations show that, if timely performed, case isolation is sufficient to contain a MARV outbreak (results are reported in Figure 3B). In particular, our analysis reveals that even low daily isolation probabilities (around 20%), if combined with first isolation day no larger than 2–3 days after symptoms onset, can drastically reduce the impact of the epidemic: the cumulative number of MARV deaths drops from 42.3% to less than 0.05% of the population. This is shown in more detail in Figure 3C, where we assume a daily isolation probability of 20%. Our results suggest that MARV epidemics can be contained if the first isolation day is less than 4 days; late interventions are not sufficient to interrupt the chain of infections and thus are able only to mitigate the epidemics. Remarkably, when isolation of cases occurs early in the individual course of the disease, the total number of isolated cases in the population is very low, with peak incidence of isolated cases of less than 2 per 10,000 individuals; therefore, the burden for the public health system would be moderate.

Figure 3D shows the possible effects of a behavioral response of the population (e.g., the avoidance of behaviors favoring disease transmission or the limitation of the exposure to environments/contexts highly suitable for MARV transmission). Model simulations suggest that a reduction of potentially infectious contacts of about 20% is sufficient to interrupt the chain of infections and thus to contain the epidemic, even if the first isolation day is larger than 3 days.

### Age–specific Susceptibility to Infection

Data on the first 124 identified cases show that about 75% of the infections occurred in individuals aged 5 years or younger [15], while the fraction of individuals of that age in Angola in 2005 was 26.4% [21]. This suggests the possibility of an existing pattern of susceptibility to infection by age. Therefore, similarly to what was done in [35], [36] for influenza, we divided the population in two age groups: children aged 5 years or younger and the rest of the population; the latter is assumed to be less susceptible to the disease of a factor 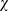. Then we parameterize this new version of the model (having two unknown parameters: the transmission rate and 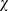) in such a way that the doubling time of the simulated epidemics is the same as observed during the 2005 outbreak in Angola and that the proportion of cases aged 5 years or less accounts for 75% of the total number of infections.

**Figure 4:**
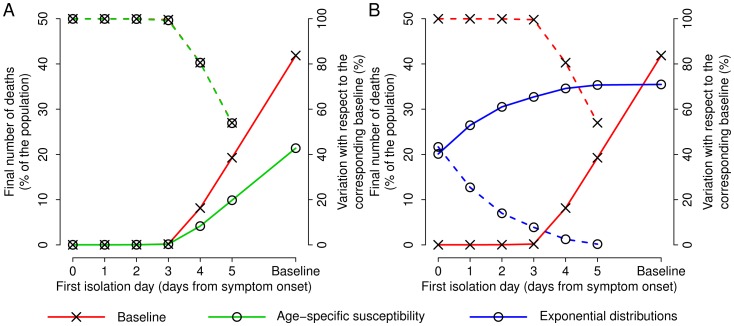
Effectiveness of social distancing strategies under alternative hypotheses. **A** Solid lines represent the final number of deaths (percentage of the population, scale on the left axis) as a function of the first isolation day. Dashed lines represent the percentage variation of the final number of deaths with respect to simulations not considering interventions (scale on the right axis). Red and cross refers to the baseline scenario (assuming the same level of susceptibility to infection in all individuals and time–variable viral load); green and circles refer to simulations where age–specific susceptibility to infection is considered. Please note that the dashed red line is almost coincident with the dashed green line. **B** As in **A** where red and cross refers to the baseline scenario; blue and circles refer to simulations where the distributions of both latent and infectious periods are assumed to be exponential with mean 6.5 days and 3 days, respectively (as in [41]). In both panels of this figure, simulations assume that the isolation of cases starts when 3 deaths per 100,000 individuals are observed in the population, the daily isolation probability is kept fixed to 20% and 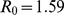, as in Figure 3 **C**.

We found that children are 14.9 times more susceptible to the disease than adults. Model simulations accounting for age–specific susceptibility to infection predict a similar timing of the epidemic, while they predict a much lower overall number of infections (24.6%, 95%CI: 24%–25.3%). Nonetheless, even if the values of the attack rate are largely different, the impact of the social distancing measures analyzed in this study is exactly the same in terms of percentage variation of the final epidemic size and number of deaths (see for instance Figure 4A).

### Exponentially Distributed Latent and Infectious Periods

In the scientific literature it has been shown that knowing the average value of the generation time is sufficient to reconstruct the timing of an epidemic (see for instance [35]–[39]). On the other hand, in order to evaluate the effectiveness of individually targeted control strategies such as isolation of cases, antiviral treatment and prophylaxis, the shape of the distribution of the generation time is crucial [22]–[24], [40]. In this section we show the differences between assuming our data–driven distribution of the generation time with respect to the assumption that both latent and infectious period are exponentially distributed, as in classical mathematical models [27]. In the latter case, we used the best available estimates of latent and infectious period as given in the literature on Marburg virus [41].

In [41], by fitting the epidemic curve of MARV cases during the epidemic in Angola and considering that the outbreak started in October 2004, the author estimates exponentially distributed latent and infectious periods having mean 6.5 days and 3 days respectively. Such values lead to a generation time of 9.5 days, which is in excellent agreement with the value of 9 days (95% CI: 8.2–10 days) found in the current study by analyzing viral load data in non-human primates. Despite the excellent agreement on the average value of the generation time, assuming different 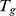 distributions results in remarkably different effectiveness of case isolation strategies, though the value of 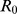 we used is the same in both scenarios (see Figure 4B). In particular, when latent and infectious periods are assumed exponential, only very quick identification and isolation of cases lead to a remarkable reduction in the number of avoided deaths.

## Discussion

In this work we estimated MARV generation time distribution and transmission potential. We estimated an average generation time of 9 days (95% CI: 8.2–10 days) and that the deaths caused by MARV infections occurred a median of 7 days (range, 5–9 days) after symptoms onset: the latter value is slightly longer than what was observed in MARV hospitalized (human) cases in the Angola outbreak. In particular, the WHO reports that most deaths have occurred between 3 to 7 days after the onset of symptoms [42], which is in agreement with the findings of [7], where most of MARV fatal outcomes occurred around day 5 from symptoms onset. Moreover, our estimate of a long tail of infectiousness in MARV cases who survive to the infection is supported by the results reported in [4], where the authors have observed positive MARV viral load 32 days after hospitalization. By relaxing the hypothesis that in MARV infected individuals death would occur at the peak of viremia and instead assuming that death probability is directly proportional to the viral load, we obtained a consistent value for the average generation time, namely 9.3 days (95% CI: 3.7–14.6 days), even if the variability became much larger. Moreover, we estimated that among individuals who die, the death occurs a median of 9 days (range, 0–56 days) after symptoms onset. According to this alternative assumption we estimated a range for fatal outcomes much closer to that observed during the Marburg hemorrhagic fever outbreak occurred in the Democratic Republic of the Congo in 1998–1999, where the estimated range was 0–70 days [43].

We estimate the basic reproduction number in the 2005 MARV epidemic in Angola to be 1.59 (95% CI: 1.53–1.66), in good agreement with the estimate of 1.62 (95%CI: 1.6–1.64) given in [41], which has been obtained by analyzing the same outbreak. Moreover, our estimate lays in between the estimates for two epidemics of another filovirus: the Ebola virus. In particular, 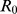 was estimated to be 1.83 (SD: 0.06) for the 1995 Ebola epidemic in the Democratic Republic of the Congo [29], and 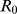 was estimated to be 1.34 (SD: 0.03) for the 2000 Ebola epidemic in Uganda [29]. In addition, we estimated that children aged 5 years or younger were about 15 times more susceptible to infection than the rest of the population. This pattern is opposite to that observed in the second largest outbreak previously recorded, which occurred in the Democratic Republic of the Congo, were only 8% of cases were aged less than 5 years [15]. This suggests that the high susceptibility to infection we found in children is mainly due to human behaviors rather than to biological processes. However, the data we used to estimate the age–specific susceptibility to infection are quite uncertain and refer only to the first phase of the epidemic. Moreover, the unusual age distribution (75% of cases aged 5 years or younger) may imply unusual circumstances - either biased surveillance or atypical exposure route (e.g. needle sharing in a pediatric ward) - difficult to account for.

Model simulations are consistent with the data observed in the 2005 MARV epidemic in Uige region, Angola. Our results suggest that, in order to reduce the impact of an epidemic, crucial factors are: *i)* timely detection of cases that can be obtained by applying clinical/epidemiological case definitions (and possibly supported by to on-site laboratory diagnosis [44]); and *ii)* massive social mobilizations (e.g., through information campaigns on risks and transmission routes of the disease). In particular, our analysis shows that a timely isolation of cases (starting no later than 2–3 days after symptoms onset, 20% daily probability of isolation) is sufficient to contain a MARV epidemic with an affordable burden for the health system. Thus, it represents a suitable intervention even when only low resources are available. Despite the effectiveness of social distancing strategies in controlling MARV outbreaks, the development of both vaccines and therapies is still crucial in order to limit the number of cases/deaths and to remarkably reduce disease severity and CFR.

Certainly, the availability of new data on MARV virology and epidemiology would be key for improving estimates of both generation time and reproduction number, and for better evaluating the effectiveness of control measures. In fact, the reduced data availability led us to introduce several approximations in our analysis, which have to be considered as study limitations. First of all, the epidemiological data on the 2005 MARV epidemic in Angola we analyzed [15] come from a secondary source of data, of which, we cannot vouch for the quality of. For instance, the adopted case definition and whether it was uniformly used through the whole course of the epidemic is unclear. Another open question is how surveillance was performed early in the outbreak, in fact the dataset is only partial: a retrospective analysis showed that the outbreak probably started in October 2004, while the first entry in the analyzed dataset dates March 2005. This suggests that, especially at the beginning of the outbreak, the actual case count could have been highly inaccurate. However, despite this lack of knowledge on the first phases of the epidemic, since the cumulative number of cases shows a clear exponential growth rate in March–April 2005, we are able to estimate the epidemic doubling time. Second, we assume that infectiousness over time is directly proportional to viral load. Unfortunately, given the scarcity of virological and epidemiological data, this assumption, though common to other studies on infectious diseases (see for instance [22]–[24], [40]) and more biologically sound than assuming constant infectiousness over time, is difficult to validate. Third, given the lack of data on viral load in human infections, we estimated infectiousness profiles over time, and thus generation time distribution, by analyzing data from a study on non-human primates. This assumes that the basic mechanism regulating within- and between- host dynamics of MARV in different primate species are similar, for instance in terms of disease severity, pathology, or kinetics of immune response. However, as we are interested in estimating the shape of the density distribution rather than absolute values of infectiousness over time, this would partially reduce the differences between species. Moreover, despite the fact that our estimate is based on the analysis of data in non-human primates, we found an average value of the generation time which is in remarkable agreement with that previously estimated in [41] through model fit to the cumulative count of MARV (human) infections in 2004–2005 in Angola. Moreover, since no precise information is available, we made a simplifying hypothesis: we assume that latent and incubation periods coincide. Another important point is that the analyzed data on viral load in non-human primates were collected in 2001, before the Angola strain of Marburg virus was discovered and there is evidence in monkeys that the pathogenicity, and thus likely the profile of viremia, from the Angola strain varies relative to other strains [45], [46]. This may explain why our estimates on the time lasting from onset of symptoms to death are in good agreement with the findings obtained by analyzing the Marburg hemorrhagic fever outbreak occurred in the Democratic Republic of the Congo in 1998–1999 [43], while they are slightly longer than those observed in the Angola epidemic [7]. Clearly this poses questions both in terms of whether modeling basis is appropriate and how extrapolatable the results are to outbreaks of other strains of Marburg virus. Given the lack of empirical data, which calls for new studies, answers to these questions are difficult to obtain. However, despite all the mentioned limitations mainly deriving from the type, amount and quality of the available empirical epidemiological/virological data, our estimates appear consistent with the scientific literature on Marburg hemorrhagic fever [4], [7], [41], [43].

In conclusion, our analysis reveals that Marburg virus is characterized by a relatively small reproduction number and by a relatively long generation time. Such factors, along with the extremely high MARV severity and fatality, represent a possible explanation of the rarity of large outbreaks in human populations. Moreover, we estimated the shape of the generation time distribution, which is essential for the evaluation of the effectiveness of individually targeted intervention strategies. Our results also support the effectiveness of social distancing measures especially of case isolation to contain or at least to mitigate a MARV epidemic outbreak. Such findings are in agreement with the general idea that infectious diseases characterized by low transmission potential and intense symptoms are the easiest to control [47]. Nonetheless, given their extremely high severity and fatality, an epidemic caused by MARV, as well as by other filoviruses like Ebola virus, would represent a serious threat for human health, especially in the absence of treatment and prophylactic measures.
